# Mapping of the sGC Stimulator BAY 41-2272 Binding Site on H-NOX Domain and Its Regulation by the Redox State of the Heme

**DOI:** 10.3389/fcell.2022.925457

**Published:** 2022-06-17

**Authors:** Garyfallia I. Makrynitsa, Aikaterini I. Argyriou, Aikaterini A. Zompra, Konstantinos Salagiannis, Vassiliki Vazoura, Andreas Papapetropoulos, Stavros Topouzis, Georgios A. Spyroulias

**Affiliations:** ^1^ Department of Pharmacy, University of Patras, Patras, Greece; ^2^ Laboratory of Molecular Pharmacology, Department of Pharmacy, University of Patras, Patras, Greece; ^3^ Laboratory of Pharmacology, Faculty of Pharmacy, National and Kapodistrian University of Athens, Athens, Greece

**Keywords:** BAY 41-2272, nitric-oxide (NO), NMR spectroscopy, soluble guanylate cyclase (sGC), H-NOX domain, cGMP (cyclic GMP)

## Abstract

Soluble guanylate cyclase (sGC) is the main receptor of nitric oxide (NO) and by converting GTP to cGMP regulates numerous biological processes. The *β*1 subunit of the most abundant, *α*1*β*1 heterodimer, harbors an N-terminal domain called H-NOX, responsible for heme and NO binding and thus sGC activation. Dysfunction of the NO/sGC/cGMP axis is causally associated with pathological states such as heart failure and pulmonary hypertension. Enhancement of sGC enzymatic function can be effected by a class of drugs called sGC “stimulators,” which depend on reduced heme and synergize with low NO concentrations. Until recently, our knowledge about the binding mode of stimulators relied on low resolution cryo-EM structures of human sGC in complex with known stimulators, while information about the mode of synergy with NO is still limited. Herein, we couple NMR spectroscopy using the H-NOX domain of the *Nostoc* sp. cyanobacterium with cGMP determinations in aortic smooth muscle cells (A7r5) to study the impact of the redox state of the heme on the binding of the sGC stimulator BAY 41-2272 to the *Ns* H-NOX domain and on the catalytic function of the sGC. BAY 41-2272 binds on the surface of H-NOX with low affinity and this binding is enhanced by low NO concentrations. Subsequent titration of the heme oxidant ODQ, fails to modify the conformation of H-NOX or elicit loss of the heme, despite its oxidation. Treatment of A7r5 cells with ODQ following the addition of BAY 41-2272 and an NO donor can still inhibit cGMP synthesis. Overall, we describe an analysis in real time of the interaction of the sGC stimulator, BAY 41-2272, with the *Ns* H-NOX, map the amino acids that mediate this interaction and provide evidence to explain the characteristic synergy of BAY 41-2272 with NO. We also propose that ODQ can still oxidize the heme in the H-NOX/NO complex and inhibit sGC activity, even though the heme remains associated with H-NOX. These data provide a more-in-depth understanding of the molecular mode of action of sGC stimulators and can lead to an optimized design and development of novel sGC agonists.

## Introduction

The nitric oxide/cyclic guanosine monophosphate (NO/cGMP) signaling pathway plays a pivotal role in numerous physiological processes, such as the establishment of a healthy homeostasis for the cardiovascular system. Hence, its impaired function has been linked to several diseases, such as pulmonary arterial hypertension (PAH), heart failure, chronic kidney disease and erectile dysfunction (Sandner et al., 2021). The physiological “receptor” of NO is the enzyme soluble guanylate cyclase (sGC), which converts GTP to cGMP (Derbyshire and Marletta, 2009; Papapetropoulos et al., 2015).

sGC is a heterodimer formed by one *α* and one *β* subunit, each with two variants, *α*1*β*1 being the most common and best described isoform found in mammals. Each subunit consists of four distinct domains: an N-terminal H-NOX (heme-nitric oxide/oxygen binding) domain, a Per/Arnt/Sim (PAS) domain, followed by a coiled-coil region and the C-terminal catalytic domain. A prosthetic heme group binds on the H-NOX domain of the *β*1 subunit *via* a covalent bond with the residue His105. The corresponding domain of *α*1 subunit is not able to bind heme and does not exhibit the features of the H-NOX protein (Derbyshire and Marletta, 2012). NO binds to the H-NOX heme-associated iron, arising electronic, dynamic and conformational changes transmitted to the catalytic domain and eliciting the activation of the sGC (Underbakke et al., 2014; Kang et al., 2019).

Impaired sGC activity can result from either heme oxidation and subsequent loss from sGC (followed by sGC degradation) or from low NO bioavailability. Both lead to dysfunction of the NO/cGMP pathway and the development of pathological conditions (Stasch et al., 2006; Sandner et al., 2021).

In the past 20 years, two classes of therapeutic molecules have been synthesized as agonists of the sGC, to be added to the old class of NO donors: NO- and heme-independent “activators” and NO-independent “stimulators” that require a reduced heme moiety is essential for their action on sGC. Activators associate with the oxidized or heme-free sGC, by occupying the same cavity with the heme and restoring the proper function of the enzyme as well as stabilizing it (Evgenov et al., 2006). Om the other hand, although stimulator molecules increase the activity of sGC in an NO-independent manner, they are also able to strongly synergize with low NO levels, a characteristic important for their therapeutic success. So far, two stimulators have been approved by the FDA: Riociguat (trade name: Adempas), to treat forms of pulmonary arterial hypertension (PAH) (Mittendorf et al., 2009; Stasch and Evgenov, 2013; Khaybullina et al., 2014) and Vericiguat (trade name: Verquvo) for reduced ejection fraction heart failure (Follmann et al., 2017; Armstrong et al., 2018). Recently, two cryoEM structures of sGC in complex with NO and either of two stimulators, Riociguat (PDB ID: 7D9R) and YC-1 (PDB ID: 7D9S) at a resolution of 3.70 Å and 3.90 Å respectively, suggested that the binding site of Riociguat and YC-1 is located between the H-NOX domain and the two coiled-coil domains (Liu et al., 2021). BAY 41-2272 is a potent sGC stimulator which can increase sGC activity by 400-fold in synergy with NO and based on preclinical evidence (Straub et al., 2001; Dunkern et al., 2007), it could be used for treatment of pulmonary fibrosis. Despite the years of studying the biochemistry and the pharmacology of the sGC stimulators, little is known about their exact mechanism of action and how NO can modulate their activity. Additionally, static Cryo-EM studies offer little knowledge about the atomic-level conformational and dynamical changes coupled with sGC’s activation by a stimulator.

In the present study, we use as a model the recombinant H-NOX domain from the *Nostoc* sp. cyanobacterium (*Ns* H-NOX) and apply NMR spectroscopy to probe the binding behavior of the sGC stimulator BAY 41-2272, in the absence of any external ligand besides the heme prosthetic group. Additionally, we exploit the unique potential of NMR to simultaneously probe conformational changes, weak binding events and changes in the electronic and coordination state of the heme and to identify the link between the redox state of the heme and the synergistic action of the stimulators with NO. Moreover, we provide functional evidence by cell-based assays to determine the impact of the heme oxidation state on the ability of BAY 41-2272 to stimulate the sGC holoenzyme.

## Materials and Methods

### Materials

All chemicals for bacterial cultures and the protein sample preparation were from Applichem. DEA-NONOate and 5-aminolevulinic acid (hydrochloride) were purchased by Cayman chemicals and used for as NO-donor and precursor of heme’s biosynthesis respectively. 1H-[1,2,4]oxadiazolo[4,3-a]quinoxalin-1-one (ODQ) (Cayman chemicals) were used as a heme-oxidizing. The sGC stimulator BAY 41-2272 was kindly provided by BAYER AG. Non-selective PDE inhibitor (isobutyl-methyl-xanthine, IBMX) and sodium nitroprusside (SNP, NO donor in cell-based assays) were purchased by Applichem. Cyclic GMP Elisa kit and micro BCA protein assay kit were purchased by Cayman chemicals and Thermo Fisher Scientific respectively.

### Protein Sample Preparation

The experimental protocol for the expression, purification and NMR sample preparation of the *Ns* H-NOX C139A variant (residues M1-D183) has been reported previously (Alexandropoulos et al., 2016). Protein concentration of the ^15^N-labeled sample for NMR titration experiments was 0.5 and 0.8 mM for the titration experiment with NO and BAY 41-2272 respectively.

### NMR Spectroscopy

All NMR experiments acquired on a Bruker Avance III HD four channel 700 MHz NMR spectrometer at 298 K, equipped with TCI 5 mm 1H/13C/15N/D-gradient probe while ^19^F NMR spectra were acquired on a Bruker Avance III HD 600 MHz NMR spectrometer equipped with BBFO probe. All data sets were processed with Topspin 3.5 software and analyzed with CARA (Keller, 2004). Backbone and side chain assignment of the heme-bound *Ns* H-NOX domain has been reported in previous study (BMRB ID: 26048) (Alexandropoulos et al., 2016).

^19^F NMR spectra were acquired with 512 scans, 1 s recycle delay and acquisition time of 0.4893355 s.

### NMR Titration Experiments

To monitor the behavior of the individual amino acids of the ^15^N-labeled *Ns* H-NOX in the presence of BAY 41-2272, we calculated the changes of their chemical shifts in ^1^H–^15^N HSQC spectra during the NMR titration experiment. The unlabeled ligand (stock: 100 mM) was added in 9 steps in order to reach excess in the protein sample. Chemical shift perturbation (CSP) values were calculated using the Eq. 1.

$$\Delta \delta_{\mathrm{ppm}}=\;\sqrt{{\left(\Delta \delta_{\mathrm{ΗΝ}}\right)}^{2}+{\left(\frac{\Delta \delta_{Ν}}{5}\right)}^{2}}$$ (1)

The threshold was defined by calculating the standard deviation *σ* and then multiplying the value by 3 (Williamson, 2013). The same approach was applied to examine the interaction of BAY 41-2272 in the presence of NO, by preparing firstly the *Ns* H-NOX/NO through NMR driven titration experiments. For the residues with significant perturbations, their CSPs were plotted as a function of BAY 41-2272 concentration and fitted into the Eq. 2 (Williamson, 2013).

Δδppm= Δδmax{([P]t+[L]t+KD)−[([P]t+[L]t+KD)2−4[P]t+[L]t]12}/2[P]t  (2)

where *Δδ*_*max*_ is the maximum shift change on saturation, [*P*]_*t*_ is the total concentration of the protein and [*L*]_*t*_ is the total concentration of the ligand.

### UV-Vis Measurements

All the measurements were carried out on Q5000 microvolume spectrophotometer (Quawell) at room temperature with sample volume 2 µl and measurement path 0.2 mm. The spectra were recorded from the proteins in a solution of 50 mM potassium phosphate and 30 mM NaCl (pH: 7).

### Cell-Based cGMP Determination

A7r5 rat aortic smooth muscle cells (RAoSM) (ATCC, Rockville, MD, United States) were cultured in 48-well clusters in DMEM growth media (GibcoTM), supplemented with 10% Fetal Bovine Serum (GibcoTM) and 1% Penicillin/Streptomycin (Biosera).

At confluence, cells were serum-starved for 2 h in media containing 0.1% BSA and were then pre-exposed for 5 min to the non-selective phosphodiesterase (PDE) inhibitor isobutyl-methyl-xanthine (IBMX, 1 mM). Then, they were treated with the sGC stimulator BAY 41-2272 at 10 μΜ or a combination of the heme-dependent NO donor sodium nitroprusside (SNP at 100 μΜ) and BAY 41-2272, for 15 min. Some of the cells that received the BAY compound or the BAY/SNP combination were also treated with the heme-oxidizing compound 1H-[1,2,4]oxadiazolo[4,3-a]quinoxalin-1-one (ODQ) (at 10 μΜ) or its vehicle control (DMSO), either 20 min before the addition of IBMX (and hence before the sGC agonists) or 2 min after the addition of the agonist(s).

Cellular cGMP content was assessed as previously described (Argyriou et al., 2021), by collecting the extracts with HCl 0.1 M, which were analyzed by a commercial ELISA kit according to the manufacturer’s instructions. The cGMP levels in each well were normalized for the respective total protein determined by a Micro BCA Protein assay kit.

## Results

### BAY 41-2272 Binds Weakly to the *Nostoc* sp. H-NOX Domain

For the NMR experiments, the H-NOX domain from cyanobacterium *Nostoc* sp. (*Ns* H-NOX) was used as model. H-NOX domains are well-conserved among different organisms, sharing common structural features. *Ns* H-NOX shares 33.86% sequence identity with *Hs* sGC *β*1-subunit H-NOX: 17 out of 27 amino acids that form the heme cavity are identical, making it the most extensively characterized among the H-NOX family domains (Makrynitsa et al., 2019; Wittenborn and Marletta, 2021).

**FIGURE 1:**
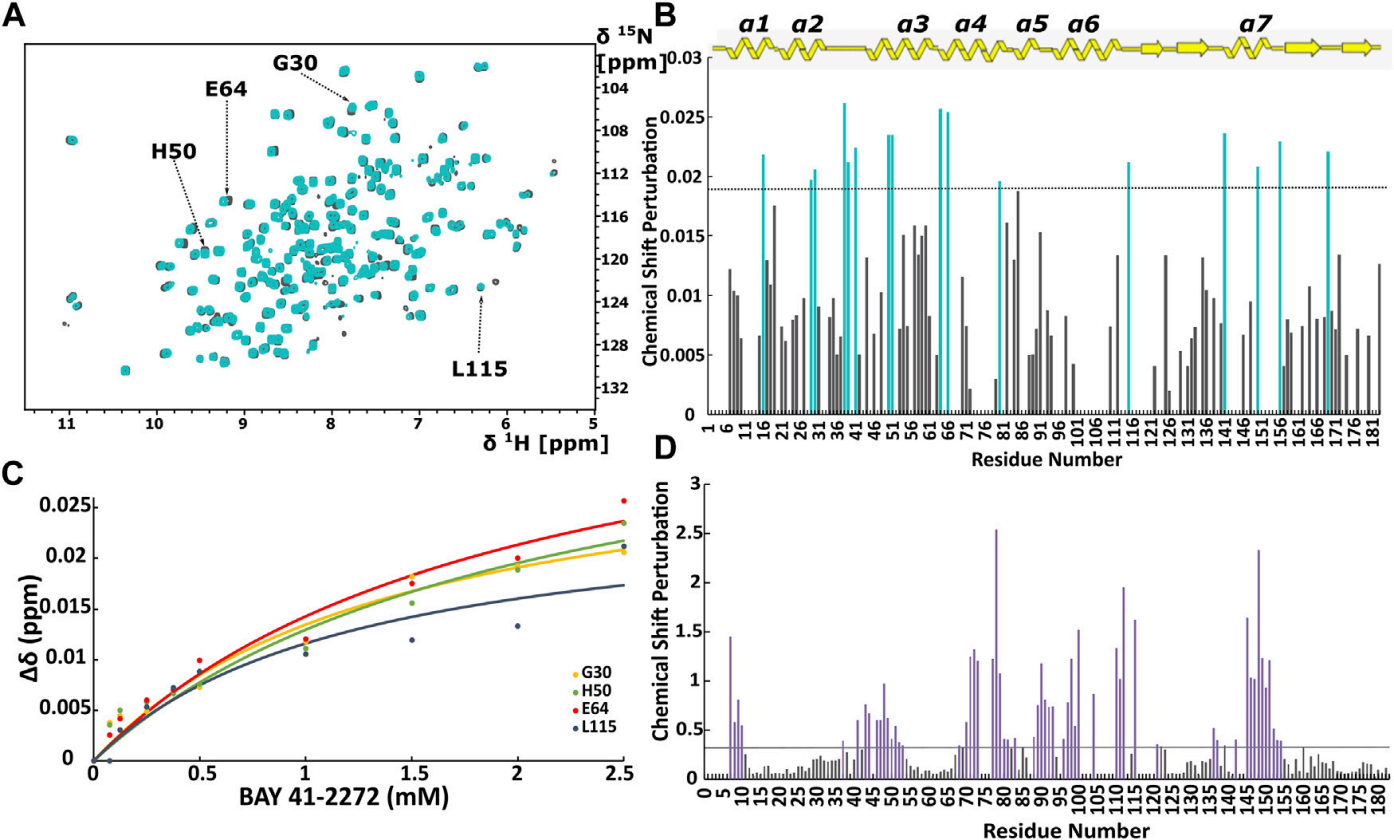
**(A)** Overlay of ^1^H-^15^N HSQC spectra of the *Ns* H-NOX domain in heme-bound state (grey) and BAY 41-2272 bound state (cyan). **(B)** Top panel: Secondary structure of X-ray structure of *Ns* H-NOX. CSP in response to BAY 41-2272 binding with threshold value 0.0188. **(C)** Plot of CSP versus BAY 41-2272 concentration; data fitted against Eq. 2. Cyan bars represent the residues with higher CSP than the threshold **(D)** CSP in response to BAY 58-2667 binding with threshold value 0.33. Purple bars indicate the residues with CSP value above the threshold after BAY 58-2667 addition (Argyriou et al., 2021).

**FIGURE 2:**
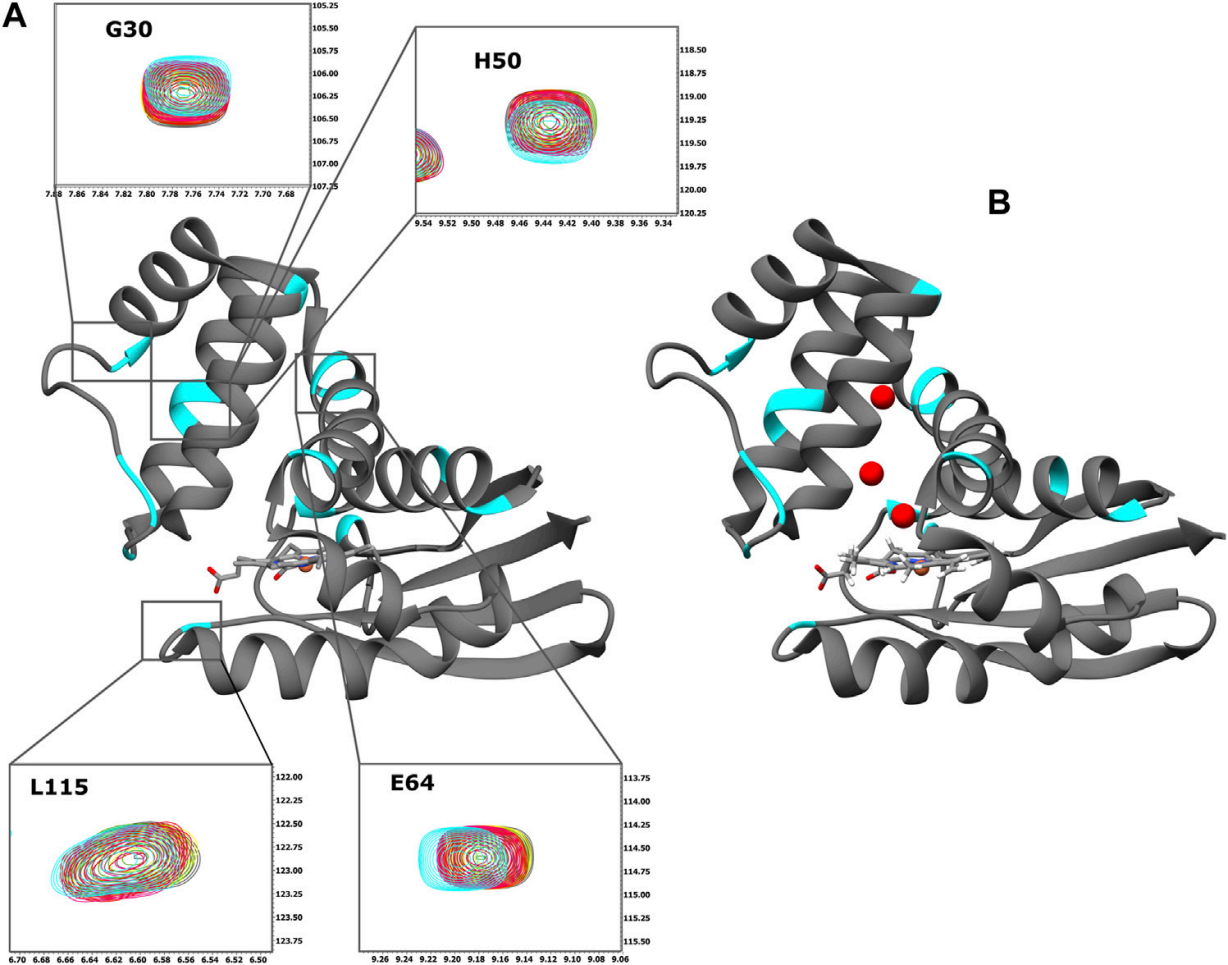
**(A)** Ribbon representation of the X-ray structure of *Ns* H-NOX domain (PDB ID: 4IAM) (Kumar et al., 2013). The residues with CSP above the threshold are mapped on the surface (cyan). Expansion of the selected HSQC regions. Overlay of HSQC spectra of *Ns* H-NOX 27 with increasing concentration of BAY 41-2272: 0 mM (grey), 0.125 mM (yellow), 0.25 mM (green), 0.5 mM (red), 1 mM (magenta), and 2,5 mM (cyan). **(B)** Ribbon representation of the X-ray structure of *Ns* H-NOX domain under 6atm of xenon gas (PDB ID: 3TFA) (Winter et al., 2011). The residues with CSP above the threshold are mapped on the surface (cyan). Ribbon representation was generated using UCSF Chimera software (Pettersen et al., 2004).

Initially, we investigated the binding behaviour of the sGC stimulator BAY 41-2272, through NMR-driven titration experiments. Overall, during the interaction, 13 residues were found in the fast-exchange regime on the NMR time-scale and displayed CSP values above threshold (Figures 1A,B, 2A). Mapping these residues on the 3D structure of the *Ns* H-NOX (PDB ID: 4IAM) (Kumar et al., 2013), shows that the amino acids affected mainly lie on the *α*_3_ and *α*_4_ α-helices as well as on the loop connecting *α*_2_-*α*_3_ and *α*_3_-*α*_4_ α-helices, are distributed on the surface of the protein and delineate a binding site juxtaposed to the heme moiety (Figures 1B, 2A). Residues defining the binding surface are conserved in other bacterial H-NOX domains (Makrynitsa et al., 2019) as well as in the *β*1 Η-ΝΟΧ domain (Supplementary Figure S1A), making it likely that they also play a role in the interaction of BAY 41-2272 with the sGC *β*1 H-NOX. Moreover, when these results are compared to the reported cryoEM structure of sGC in complex with NO and Riociguat (Liu et al., 2021), it is evident that the residues participating in the binding of the drug are the same in both cases, strengthening the evidence that BAY 41-2272 and related stimulators bind to a conserved pocket in the H-NOX domain (Supplementary Figure S1B).

CSP values of all 16 perturbed residues were plotted against the BAY 41-2272 concentration, yielding the binding constant (*K*_*D*_) for each residue (Figure 1C; Supplementary Table S1). Values of the *K*_*D*_ are very similar to the corresponding ones for *Shewanella woodyi* (*Sw*), indicating that BAY 41-2272 exhibit the same affinity towards *Ns* H-NOX domain as the IWP-051 to *Sw* H-NOX (Wales et al., 2018). It is observed a low affinity interaction (∼mM) between *Ns* H-NOX domain BAY 41-2272. However, the stoichiometry of the binding cannot be calculated because the titration experiment was performed up to 1:5. In fact, according to CSP data, BAY 41-2272 interacts with *Ns* H-NOX domain *via* one specific site on the protein surface, while the cryoEM structure of the sGC in complex with another stimulator (Riociguat) indicate a single binding site (Liu et al., 2021). Therefore, it is speculated that *Ns* H-NOX domain binds one molecule of BAY 41-2272.

Mapping of the residues with a CSP value above the threshold on the X-ray structure of *Ns* H-NOX domain under 6atm of Xenon (PDB ID: 3TFA) (Winter et al., 2011), revealed that the binding site of the BAY 41-2272 is located near the Xe atoms in juxtaposition with the two tunnels that are thought to allow the flux of NO (Figure 2B). This system of tunnels behaves as a “pathway” for NO diffusion, while mutations of these residues which result to blockade of the channels can affect the NO-sensing ability of sGC (Winter et al., 2011). The binding site of BAY 41-2272 suggests that BAY 41-2272 may serve as a block to these tunnels and prevent the escape of the NO.

When we compare the present data with those probing the interaction of *Ns* H-NOX with sGC activators (Argyriou et al., 2021) (Figure 1D), we can observe several major differences. Activators of sGC (BAY 58-2667 and BAY 60-2770) form very stable complexes with the *Ns* H-NOX domain, reducing considerably the dynamic properties of the protein and they interact mainly with H-NOX residues around the heme cavity, since they replace the heme by occupying the same space (Supplementary Figure S2). Furthermore, BAY 41-2272 exhibits much lower CSP values than those seen with BAY 58-2267, implying that the interaction of BAY 41-2272 with the *Ns* H-NOX domain is considerably weaker than that of BAY 58-2267, and implicates a completely different site of the protein, which is located on the protein surface.

### Nitric Oxide Facilitates H-NOX Domain’s Interaction With BAY 41-2272

**FIGURE 3:**
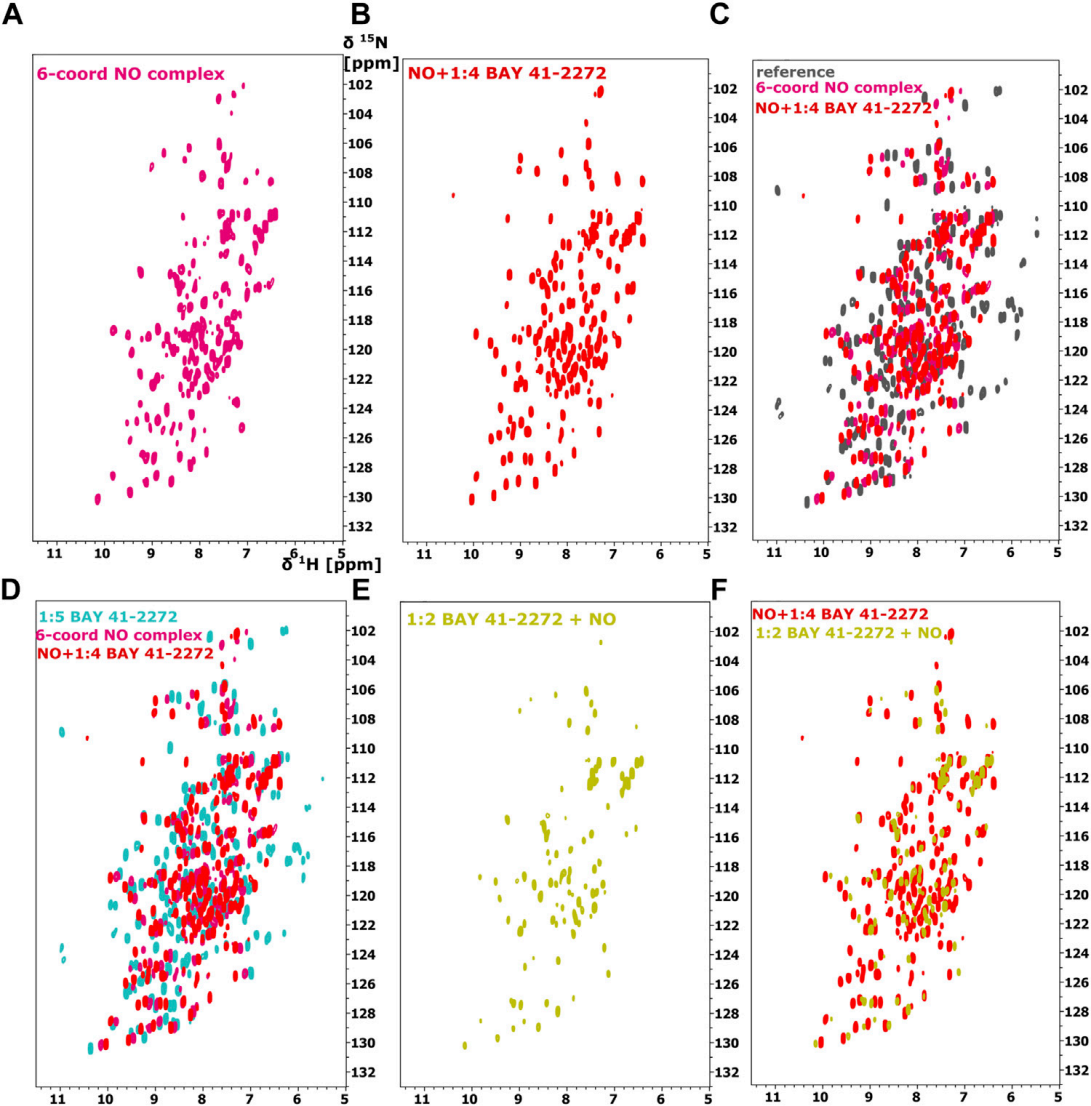
^1^H-^15^N HSQC spectra of the *Ns* H-NOX domain **(A)** in 6-coordinated NO-bound state (magenta), **(B)** with NO and 1:4 BAY 41-2272 (red), **(C)** Overlay of ^1^H-^15^N HSQC of *Ns* H-NOX domain (reference-grey) with NO (magenta) and NO/1:4 BAY 41-2272 (red). **(D)** Overlay of ^1^H-^15^N HSQC spectra of *Ns* H-NOX domain with 1:5 BAY 41-2272 (cyan), NO (magenta) and NO/1:4 BAY 41-2272 (red), **(E)** ^1^H-^15^N HSQC spectra of the *Ns* H-NOX domain with 1:2 BAY 41-2272 and NO (yellow), **(F)** Overlay of ^1^H-^15^N HSQC spectra of *Ns* H-NOX domain with NO/1:4 BAY 41-2272 (red) and 1:2 BAY 41-2272/NO (yellow).

**FIGURE 4:**
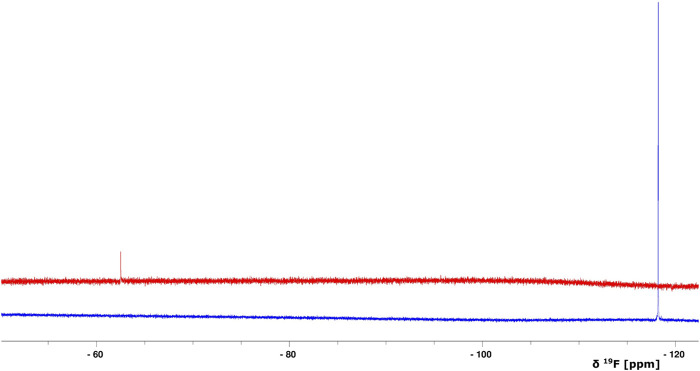
Overlay of 1D ^19^F NMR spectra of BAY 41-2272 (only the compound, no protein is added) (blue) and after the interaction with the complex *Ns* H-NOX/NO (red). The S/N ratio of the two spectra is different due to the intensity scale of the red one which is set *2.

Because stimulators are able to trigger sGC’s function in a heme-dependent manner and strongly synergize with NO (Follmann et al., 2013; Breitenstein et al., 2016), we performed NMR titration experiments of *Ns* H-NOX domain with BAY 41-2272 in the presence of NO. We prepared an *Ns* H-NOX/NO complex by adding 0.7 mM of the NO donor DEA-NONOate and then added excess BAY 41-2272, at a protein-ligand ratio of 1:4. By a look at the ^1^Η-^15^N HSQC NMR spectrum we can see the formation of a new *Ns* H-NOX domain complex, containing the heme group as well as two extra ligands, NO and BAY 41-2272 (Figure 3B). This NMR spectrum is very different compared to the spectra of the *Ns* H-NOX in the reference state (without any extra ligand besides the heme) and to the *Ns* H-NOX/NO complex (Figures 3A,C,D), yielding that low concentration of NO may increase the binding affinity of the BAY 41-2272. Unfortunately, due to the low stability of the H-NOX/NO/BAY complex, acquisition of 3D NMR spectra for the backbone assignment was not feasible. To gain more detailed insight into the interaction of *Ns* H-NOX with BAY 41-2272 in the presence of NO, and in order to confirm that the changes in the ^1^H-^15^N HSQC are indeed a consequence of the interaction with BAY 41-2272, we performed ^19^F-NMR analysis. Overlay of the two spectra, the first resulting from the addition of BAY 41-2272 alone and the second after the interaction of BAY 41-2272 with the *Ns* H-NOX/NO complex, shows a clear change on the chemical shift of the one fluorine atom of BAY 41-2272 (Figure 4) indicating that the new complex indeed results from the interaction with the BAY 41-2272 and cannot be attributed simply to a more effective interaction of the *Ns* H-NOX domain with the NO forming a more stable H-NOX/NO complex.

To test whether NO has an impact on the weak binding of BAY 41-2272, we titrated DEA-NONOate (final concentration 0.6 mM), at a ratio of 1:1 (protein/DEA), into the *Ns* H-NOX/BAY 41-2272 complex. The ^1^H-^15^N HSQC spectrum (Figures 3E,F) is different than the spectrum of *Ns* H-NOX/NO/BAY 41-2272 complex and shows that NO still interacts with *Ns* H-NOX independently of BAY 41-2272 suggesting that the action of NO excels the action of BAY 41-2272. Therefore, the weak binding of the stimulator cannot be enhanced by the subsequent addition of the NO, but is strengthened only if NO has previously formed a complex with the *Ns* H-NOX domain, as observed by UV-vis and NMR spectroscopies.

### Heme’s Oxidation State Regulates the Binding of BAY 41-2272

**FIGURE 5:**
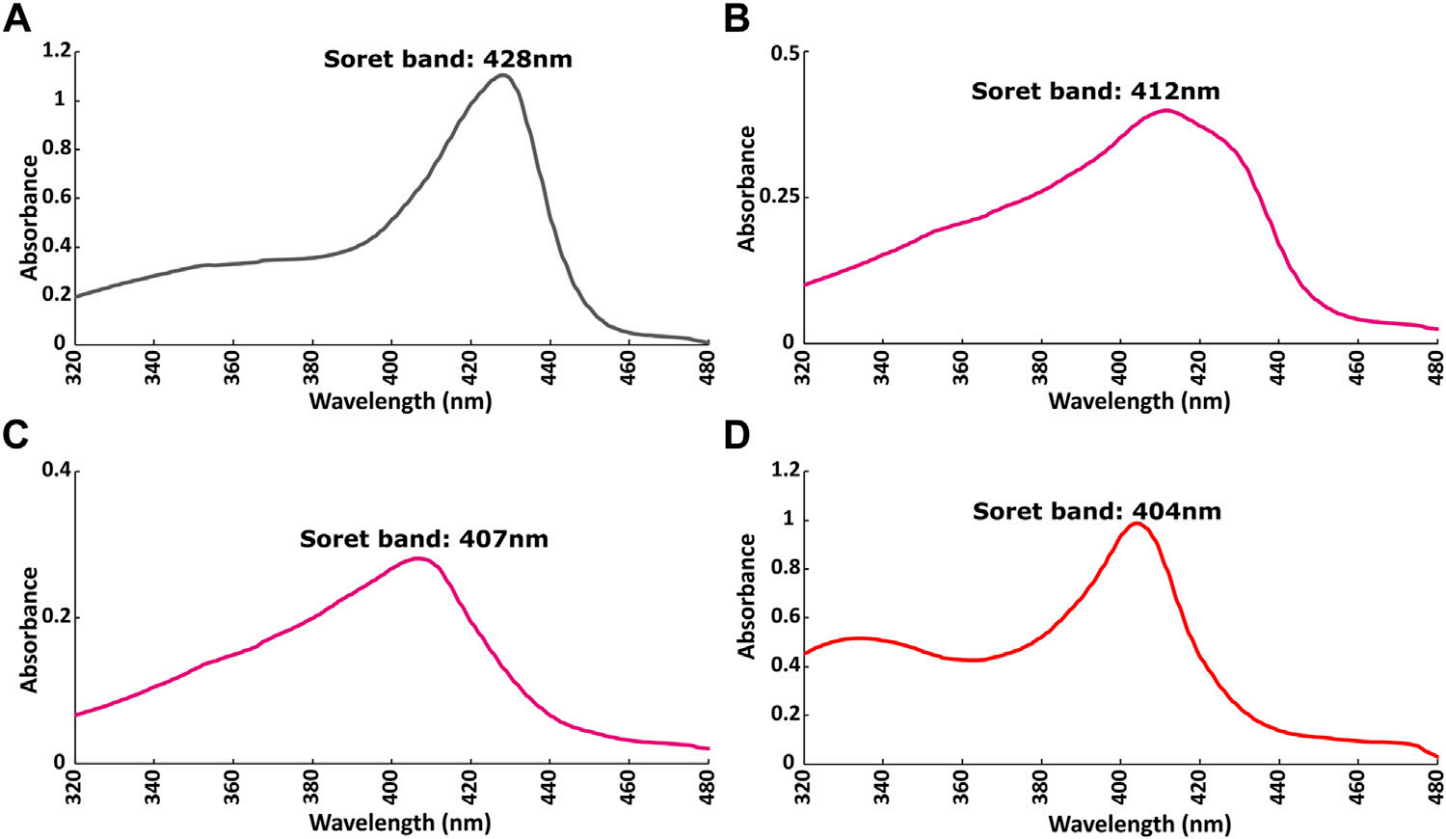
Electronic absorption spectra of *Ns* H-NOX domain **(A)** before and **(B)** after adding 0.7 mM DEA-NONOate or **(C)** 1.5 mM DEA-NONOate and **(D)** after adding 1:4 BAY 41-2272 in the presence of 0.7 mM DEA-NONOate.

The redox state of the heme moiety of *Ns* H-NOX domain was monitored by UV-vis. *Ns* H-NOX with the heme moiety in a reduced unliganded state (i.e., in the absence of NO), has a characteristic Soret band at 428 nm (Figure 5A). On the other hand, *Ns* H-NOX after addition of 0.7 mM DEA-NONOate exhibits a Soret band at 412 nm (Figure 5B), corresponding to an intermediate state consisting mainly of 6-coordinated Fe(II)-NO complex which is inactive, while 1.3 mM DEA-NONOate results in the formation of the final state of the NO complex with 5-coordinated Fe(II)-NO and a Soret band near 407 nm. Addition of BAY 41-2272 to the 6-coordinated NO complex causes a shift of the Soret band from 412 to 404 nm (Figure 5D). This value, therefore, is much closer to the value of the 5-coordinated Fe(II)-NO complex, in good agreement with published evidence (Ma et al., 2007; Tsai et al., 2010) (Figure 5C). Electronic absorption spectra indicate that BAY 41-2242 manages to lead the *Ns* H-NOX/NO complex to the active, a 5-coordinated state, even at low NO concentrations. Taken together, the NMR and electronic absorption spectra indicate that in the presence of NO, BAY 41-2272 exhibits a different type of interaction with the *Ns* H-NOX domain, imposing possibly greater conformational changes while it also impacts the heme’s environment and iron’s electronic structure. Similar behavior has been previously observed with the prototype sGC stimulator, YC-1: addition of YC-1 to the previously formed sGC-CO complex affected the electronic absorption spectra of the complex by shifting the Soret peak from 424 to 420 nm (Kharitonov et al., 1999).

### BAY 41-22 Prevents Loss of the Heme Group by Subsequent Addition of ODQ

It is now well accepted that reduced heme is necessary for the sGC-stimulating activity of stimulator-type molecules, as the oxidation of heme abolishes their activity (Stasch and Hobbs, 2009). To test the effect of heme’s oxidation after the interaction of H-NOX with BAY 41-2272, we titrated excess of the sGC inhibitor, the heme oxidant ODQ, to the *N*s H-NOX domain and then to the complex of *Ns* H-NOX with NO and BAY 41-2272.

**FIGURE 6:**
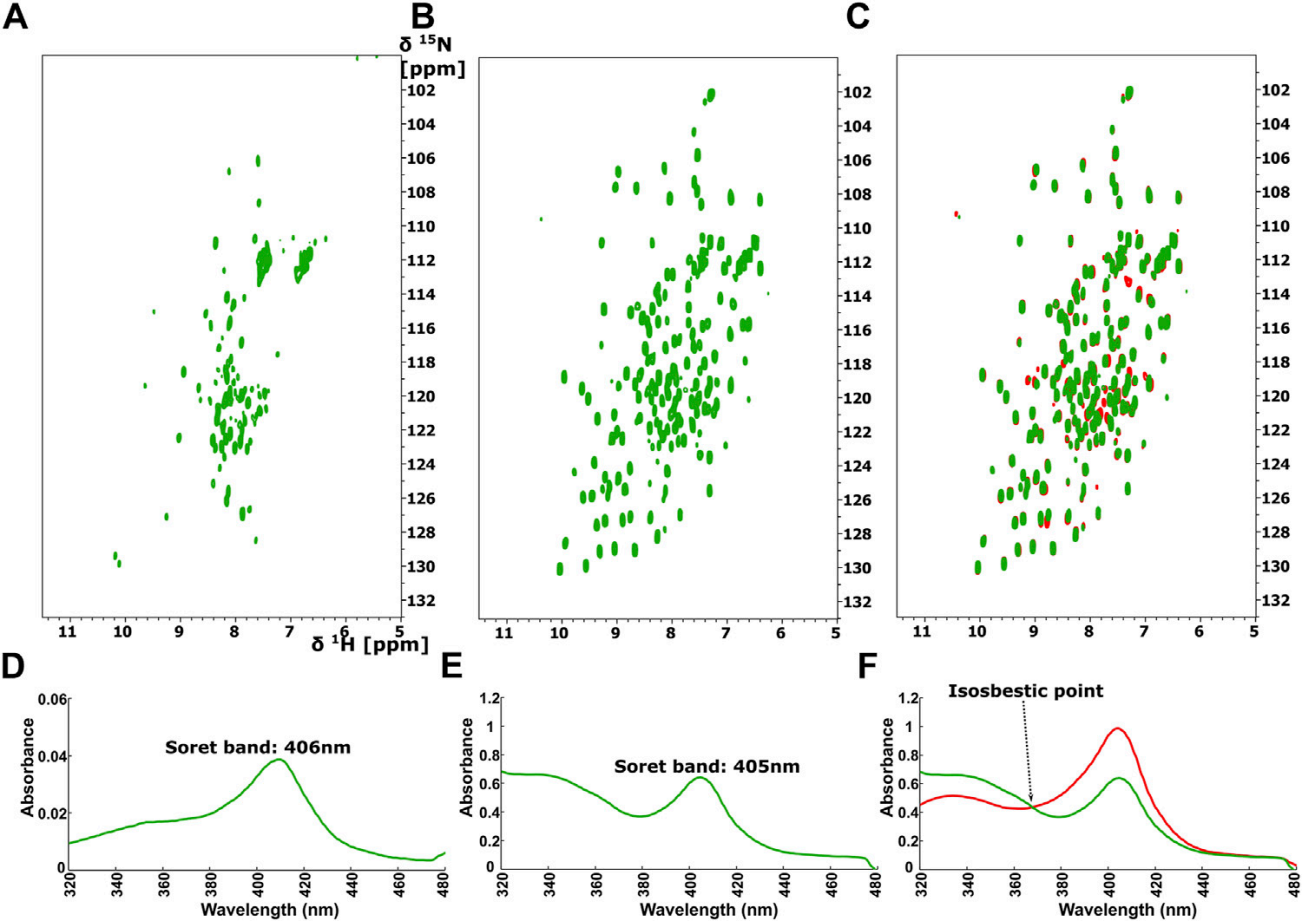
^1^H-^15^N HSQC after addition of ODQ with ration 1:1 to **(A)** the *Ns* H-NOX and to **(B)** the *Ns* H-NOX/NO/BAY 41-2272. **(C)** Overlay of ^1^H-^15^N HSQC spectra of the *Ns* H-NOX/NO/BAY 41-2272 complex before (red) and after ODQ addition (green). Electronic absorption spectra of **(D)** *Ns* H-NOX domain after addition of ODQ with ratio 1:1 and **(E)** *Ns* HNOX/NO/BAY 41-2272 after adding ODQ **(F)** Overlay of UV-vis spectra of Ns H-NOX/NO/BAY 41-2272 complex before (red) and after addition of ODQ (green) with isosbestic point at 368 nm.

The ^1^H-^15^N HSQC spectrum of *Ns* H-NOX domain, after addition of ODQ at a protein-ODQ ratio of 1:0.5, reveals that several peaks are missing. Mapping these peaks on the X-ray structure of *Ns* H-NOX domain (Supplementary Figure S3), shows that most of these peaks are located around the heme cavity, implying that ODQ is able to oxidize the heme and affect its environment even in such a low ratio. Increasing the protein-ODQ ratio to 1:3 changes completely the NMR spectrum, causing a disappearance of almost all the peaks, compatible to an unfolding of the protein (Figure 6A), with simultaneously oxidation of the heme (Figure 6D).

Surprisingly, ODQ exhibits a different behavior towards the complex of *Ns* H-NOX with NO/BAY 41-2272. Unexpectedly, addition of ODQ did not alter the ^1^H-^15^N HSQC spectrum (Figures 6B,C), suggesting that ODQ does not cause any detectable conformational changes. However, we can observe a small but distinct shift of the Soret band from 404 to 405nm, while the absorbance is significantly lower after the addition of ODQ (from 0.9844 to 0.6395) (Figure 6E), implying loss of the heme’s amount. These data suggest that ODQ is still able to oxidize the H-NOX-bound heme but that the binding BAY 41-2252 prevents the loss of the heme moiety and therefore allowing the *Ns* H-NOX domain to retain its structure.

### Varying the Timing of Heme Oxidation: Effect the sGC Catalytic Function

The synergy of sGC stimulators such as BAY 41-2272 with NO and its dependence on the presence of heme are well-described characteristics (Friebe et al., 2020). In A7r5 RAoSM cells, which naturally express the soluble guanylate cyclase (sGC) *α*1*β*1 isoenzyme (Holt et al., 2016), BAY 41-2272 is capable of increasing cGMP levels by itself and synergizes with the NO donor sodium nitroprusside (SNP) (Supplementary Figure S4), indicating that our assay system works as expected based on the stimulator’s mechanism of action (Adderley et al., 2012).

**FIGURE 7:**
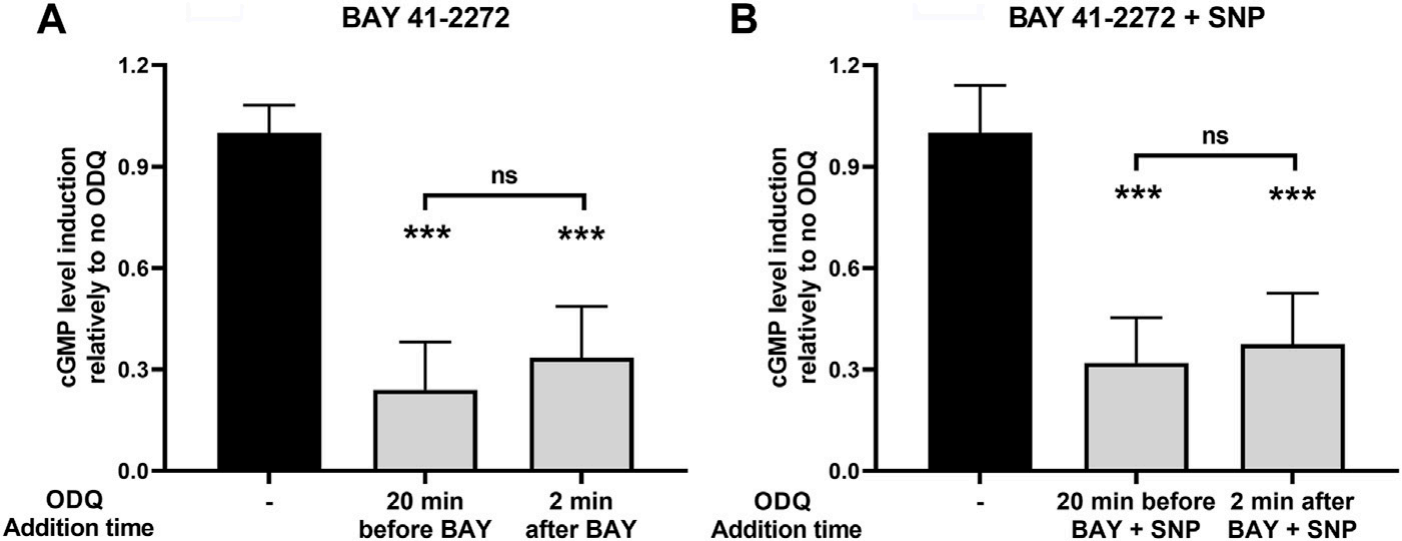
The effects of ODQ after sGC activation by **(A)** the sGC stimulator BAY 41-2272 (10 μΜ) alone or **(B)** in combination with the NO donor SNP (100 μΜ), in A7r5 rat aortic smooth muscle cells. The heme oxidant ODQ (10 μΜ) or its vehicle (DMSO) was added either 20 min before or 2 min after the sGC agonists. 15 min after the exposure to the agonists, cell extracts were taken, analyzed for cGMP content and normalized for total protein. Data are expressed as means ± STDEV of *n* = 4 determinations, obtained in two independent experiments. *p* values were determined by 2-tailed Student’s *t*-test. **(A)**. The effect of BAY 41-2272 in the absence of ODQ was taken as 1.0. ***: *p* < 0.001 relative to BAY 41-2272 treatment in the absence of ODQ. **(B)**. The effect of BAY 41-2272 + SNP in the absence of ODQ was taken as 1.0. ***: *p* < 0.001 relative to BAY 41-2272 + SNP treatment in the absence of ODQ.

Most *in vitro* studies on the role of heme on sGC function are focused on examining the role of prior heme oxidation (and loss from H-NOX) on the effectiveness of heme-dependent sGC agonists (Morbidelli et al., 2010; Chen et al., 2020). We attempted to address the NMR-based results shown above by testing whether an oxidant such as ODQ can interfere with the sGC stimulatory activity, after the BAY/sGC *β*1 complex has been allowed to form. To do so, we added the oxidant ODQ 2 min after BAY 41-2272 addition and compared its effect side-by-side with the increases in cGMP formation obtained when cells were pretreated with ODQ 20 min before BAY 41-2272. Our results indicate that ODQ is capable of significantly reducing the effect of BAY 41-2272 to a similar extent when added either prior to (↓ 76% ± 14%) or after (↓ 67% ± 15%) the sGC agonist (Figure 7A).

Furthermore, we addressed the same question using sGC activation caused by the combination of BAY 41-2272 and SNP. Similarly, we observed that ODQ treatment 2 min after co-stimulation with BAY 41-2272 + SNP was able to reduce cGMP levels (↓ 63% ± 15%), i.e., to a similar extent to that obtained with ODQ pretreatment (↓ 68% ± 13%) (Figure 7B).

## Discussion

The study of the interaction of the sGC stimulators with the H-NOX domain allows us to determine in unprecedented detail the molecular mode of interaction of this class of drugs with H-NOX, by providing insights into 1) the affinity of binding, 2) the H-NOX residues involved and 3) how the formation of an active complex consisting of the H-NOX domain and the stimulator compound can be impacted by the redox-state of the heme and the addition of NO.

The available cryo-EM structures of sGC with a stimulator (Riociguat or YC-1), although quite informative, are characterized by low resolution. For the first time, we monitored how BAY 41-2272 interacts with the H-NOX domain with and without the synergistic presence of NO, in real-time, by solution NMR. Our NMR data reveal that BAY 41-2272 interacts rather weakly with a specific site of the H-NOX domain in the absence of NO. The amino acids participating in the interaction are located mainly on the surface of the protein, so they are not part of the heme cavity. The interaction surface includes regions of the *α*_3_ and *α*_4_ α-helices as well as the loop connecting *α*_2_-*α*_3_ and *α*_3_-*α*_4_ helices. Importantly, these residues are close to the tunnels permitting the flux of diatomic gas agonists inside the protein where they elicit its stimulation. Our findings suggest that BAY 41-2272 may act as a gatekeeper at this tunnel network to prevent the gas molecules to diffuse outside the protein, thus potentiating their effect.

BAY 41-2272 exhibits a strikingly different binding behaviour compared to that of the activator BAY 58-2667. While BAY 41-2272 interacts weakly on the surface of the *Ns* H-NOX domain juxtaposed to the heme cavity, BAY 58-2667 occupies the heme cavity and affects mainly regions close to heme located on the *α*5, *α*6, and *α*7 helices, forming a new, very stable complex and erasing completely the dynamic properties of the system (Argyriou et al., 2021). The different interaction mode of stimulators and activators may therefore justify the different impact they have on the activation of the sGC enzyme.

So far, there is no X-ray structure available for the H-NOX/BAY 41-2272 complex, probably due to the compound’s weak binding, with a *K*_*D*_ that we determined is at the mM range. However, in the presence of NO, BAY 41-2272 exhibits a quite different binding behaviour: its binding is significantly enhanced, compatible with its strong synergistic action with NO. Our cell-based assays show that BAY 41-2272 stimulates sGC activity almost 15-fold above baseline, while BAY 41-2272 along with SNP (NO donor) can activate the enzyme by 80-fold (Supplementary Figure S2). The *K*_*D*_ we determined for the interaction of BAY 41-2272 with the *Ns* H-NOX is somewhat higher compared to that observed for the interaction of the stimulator IWP-051 with the H-NOX domain from *Shewanella woodyi* in the presence of carbon monoxide (*K*_*D*_ = 1.9 mM) (Wales et al., 2018). We conclude that stimulators of sGC tend to interact weakly with the H-NOX domain of various organisms and use similar protein residues. These findings along with the information derived from the 3D structures of sGC, strongly imply that in spite of the presence of the other sGC domains (PAS, CC, CD), the H-NOX domain and the associated heme play a pivotal role in the stimulation of the enzyme’s activity as the primary sensor of both NO and stimulator molecules. This conclusion is further supported by the NMR and UV-visible data presented here which illustrate the synergistic action between NO and stimulators, clearly implicating the H-NOX domain as the main drug target for the regulation of sGC activity, whether it is manifested as impairment or enhancement (Liu et al., 2021).

NO plays a critical role, with the exact mechanism of this interaction still not entirely elucidated. Superimposition of the free and NO-bound *Ns* H-NOX domains shows no significant alterations regarding the overall arrangement (Makrynitsa et al., 2019), suggesting that the H-NOX-mediated activation of sGC may be driven by protein dynamics and redox changes, rather than large conformational changes of the H-NOX domain itself. This, of course, cannot exclude changes in the orientation of the sGC domains or even domain rearrangements, for example, if the stimulator binds on the interface of H-NOX with other sGC domains. Furthermore, the binding of BAY 41-2272 may be enhanced by NO thanks to changes in the redox state of the heme induced by NO. The results presented herein indicate that the *Ns* H-NOX domain forms a folded 6-coordinated Fe(II)-NO complex with a characteristic Soret band at 412 nm. BAY 41-2272 favours the transition of the previous complex to the 5-coordinated Fe(II)-NO complex with a Soret band at 405 nm, even without excess amount of NO. In contrast, the interaction with NO is not appreciably affected by the presence of BAY 41-2272, since NO alone can form a complex with the *Ns* H-NOX domain. In essence, the action of BAY 41-2272 is enabled and potentiated by NO and not *vice versa*. Cooperation of BAY 41-2272 with NO results in the formation of a unique complex with the *Ns* H-NOX domain, where the Fe of the heme adopts a different electronic structure, either through redox switch or through the differentiation of the coordination geometry, which coincides with a fully active state of the protein. ΒΑΥ 41-2272, therefore, allows the *Ns* H-NOX domain to reach more easily and efficiently the fully active state with low NO concentrations. Our findings along with our previous results (Argyriou et al., 2021) put forth the paramount importance of the oxidation state of the heme for the effective action of sGC agonists and provide a possible mechanism of action for stimulators.

Furthermore, NMR results reveal that the complex *Ns* H-NOX/NO/BAY 41-2272 is overall insensitive to the action of ODQ, a selective and irreversible inhibitor of sGC. Taken together with the observation of an isosbestic point at 369 nm (Figure 6F), these results indicate that the ODQ may convert the ferrous heme/NO complex to a new complex with oxidized heme, without any intermediate state. This particular complex exhibits the same conformation with the *Ns* H-NOX/NO/BAY 41-2272 complex but with the exception of the oxidized heme. Our interpretation is that BAY 41-2272 may trap the oxidized heme and unbound NO inside the protein, securing the conformation of the *Ns* H-NOX domain and hindering the exit of the oxidized heme.

Our cell-based assays show that, when sGC activation is assayed in cells that carry the mammalian holoenzyme, the prior formation of a heme/NO/BAY 41-2272 complex still allows ODQ to reach in, oxidize the heme and thus interfere with sGC activation. Similar results (K. Salagiannis, unpublished observations) were obtained by allowing more time (10 min) for the complex of sGC and stimulators (SNP + BAY) to form before ODQ addition. These observations are compatible with our structural NMR results where the *Ns* H-NOX/NO/BAY 41-2272 complex appears to maintain its conformation despite the action of ODQ, since the ferric heme remains associated with the protein but without any NO molecules bound.

In the present work, we point out the crucial role of the reduced heme for the proper function of the sGC enzyme during the stimulation by NO and BAY 41-2272. ODQ manages to inhibit the sGC-driven cGMP formation by oxidizing the heme while simultaneously BAY 41-2272 protects the conformation of the H-NOX domain. Hence, we are led to conclude that the catalytic activity of sGC may not be guaranteed by the simple presence of heme in the H-NOX cavity, but that its coordination state and relation to the attached Fe or the stereochemistry of the Fe-heme association may play a paramount role.

Overall, we describe an analysis in real time of the interaction of the sGC stimulator, BAY 41-2272, with the recombinant *Ns* H-NOX, map the amino acids on the protein surface that mediate this interaction and provide evidence to explain the characteristic synergy of BAY 41-2272 with NO: prior association of NO with the heme plays a permissive role in BAY’s association, while the presence of BAY 41-2272, in return, allows *Ns* H-NOX to be sensitive to low NO levels, by establishing a fully reduced heme prosthetic group and thus maximal sGC activity.

## Data Availability

The original contributions presented in the study are included in the article/Supplementary Material, further inquiries can be directed to the corresponding author.
