# Supplementary figures and images for: Mapping of the sGC Stimulator BAY 41-2272 Binding Site on H-NOX Domain and Its Regulation by the Redox State of the Heme
Source: Front Cell Dev Biol. 2022 Jun 17;10:925457. doi: 10.3389/fcell.2022.925457 (PMC9247194; doi:10.3389/fcell.2022.925457)

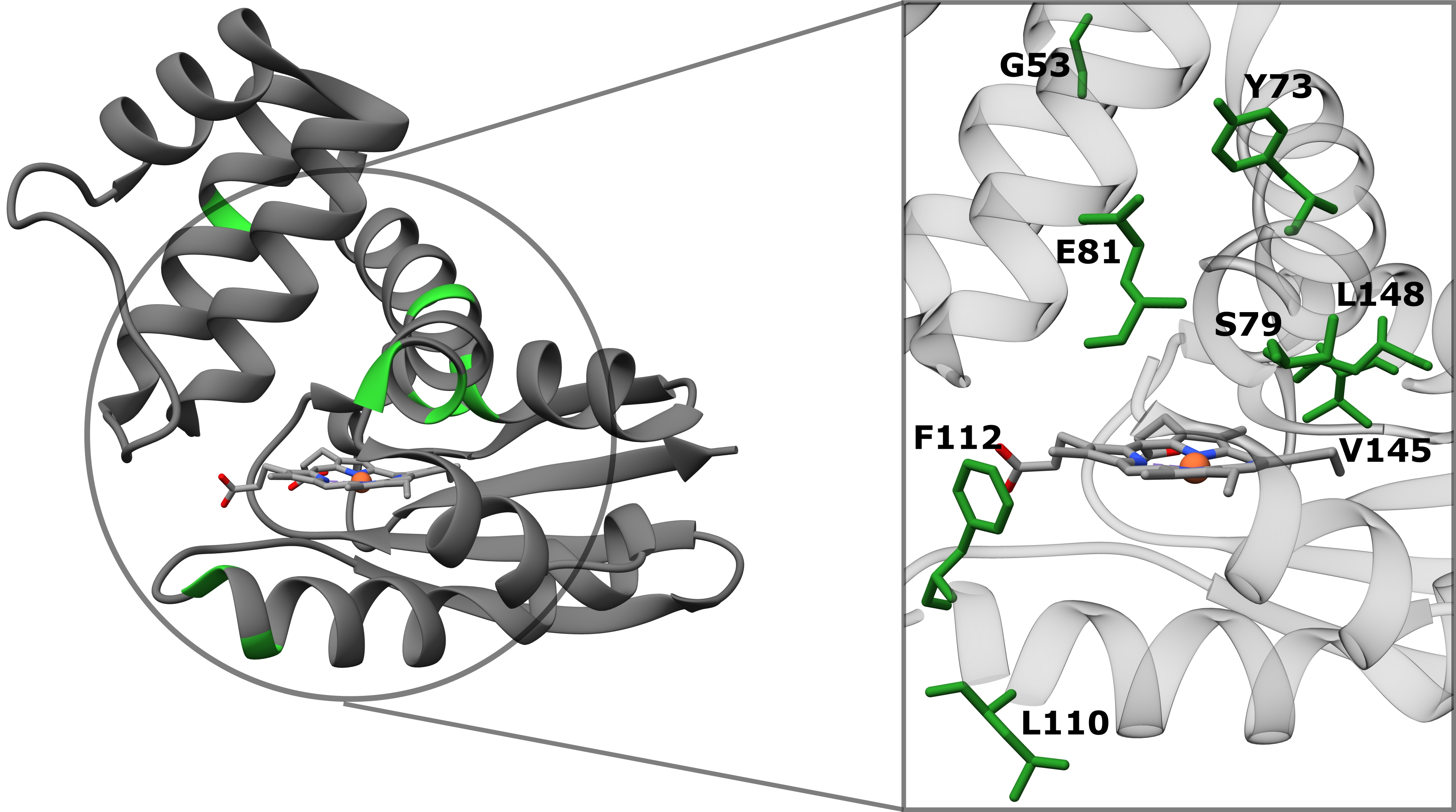

Supplement: Supplementary file 1 [file Image3.JPEG]

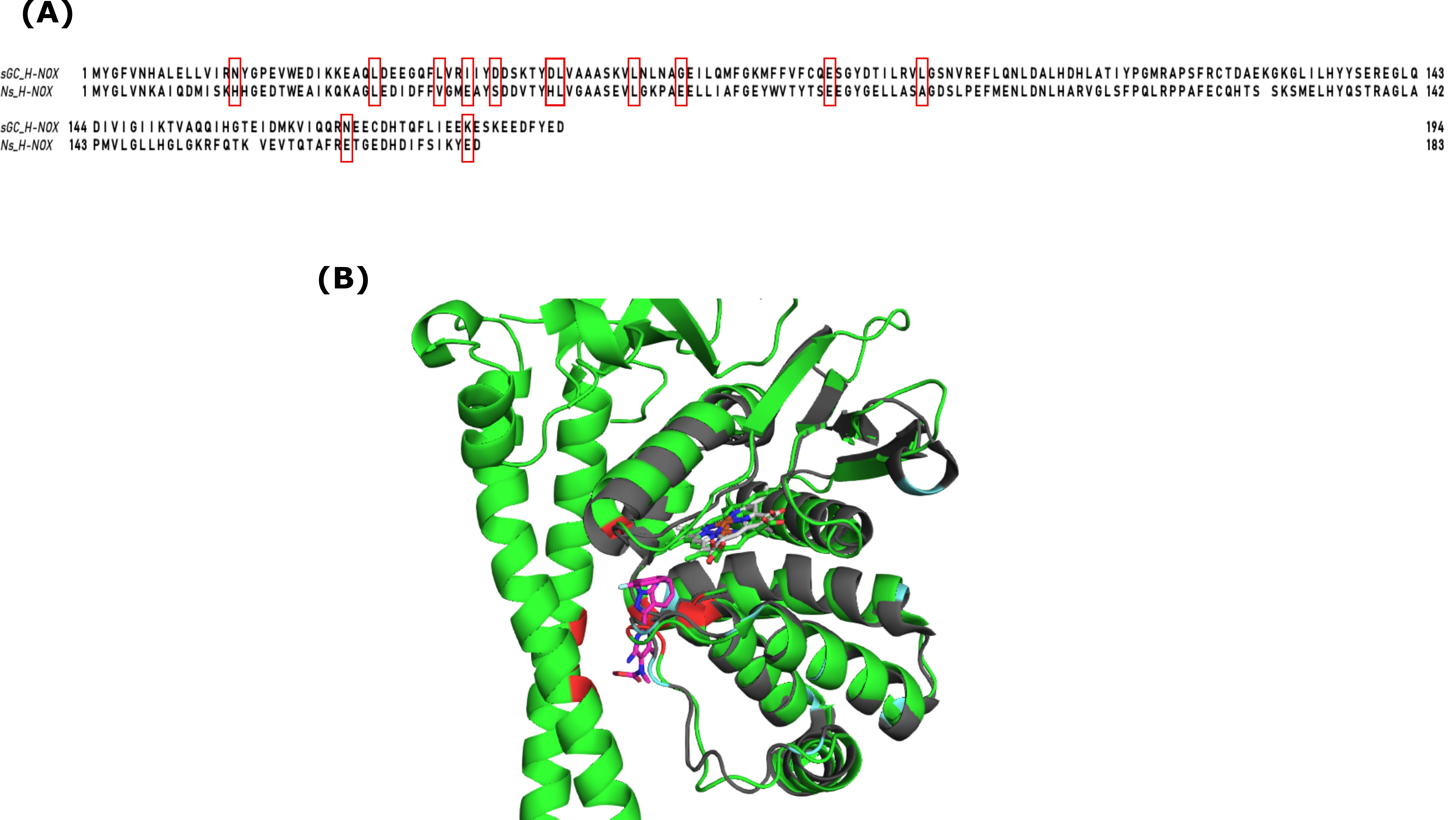

Supplement: Supplementary file 3 [file Image1.JPEG]

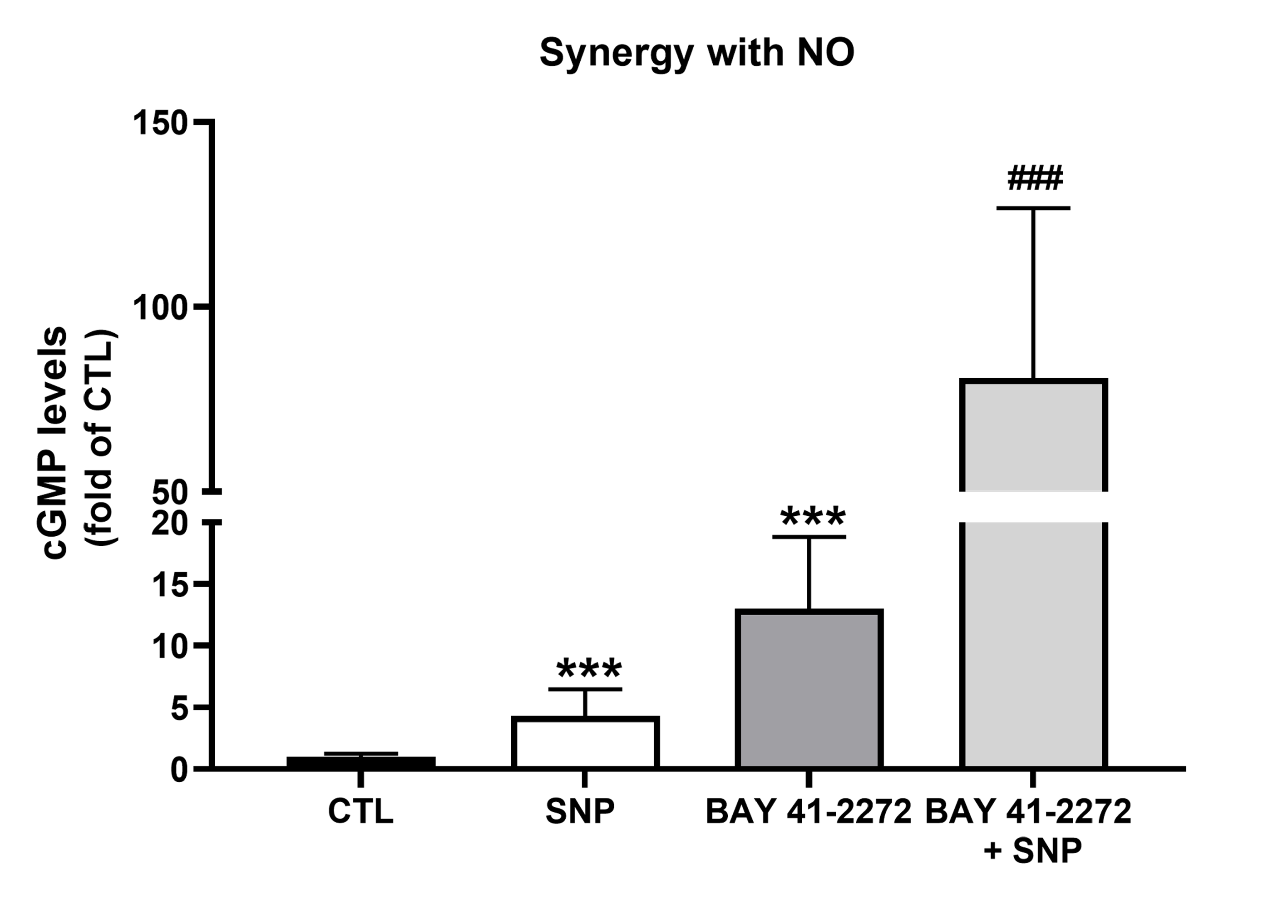

Supplement: Supplementary file 4 [file Image4.JPEG]

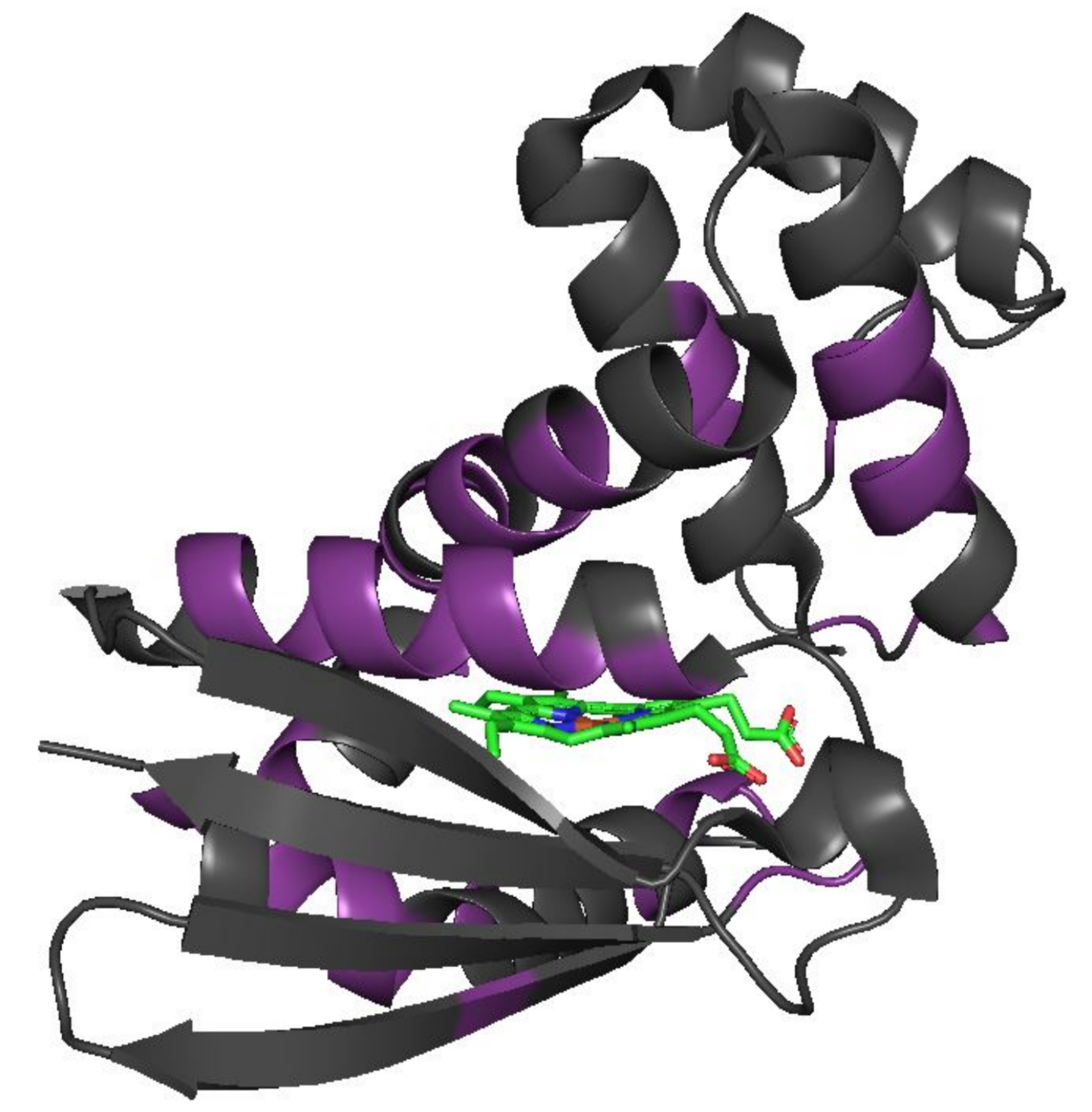

Supplement: Supplementary file 5 [file Image2.JPEG]
