# Supplementary material for: Mapping of the sGC Stimulator BAY 41-2272 Binding Site on H-NOX Domain and Its Regulation by the Redox State of the Heme
Source: Front Cell Dev Biol. 2022 Jun 17;10:925457. doi: 10.3389/fcell.2022.925457 (PMC9247194; doi:10.3389/fcell.2022.925457)
Supplement: Supplementary file 2 [file Table1.DOCX]

Supplementary Material

# Supplementary Figures and Tables

## Supplementary Figures


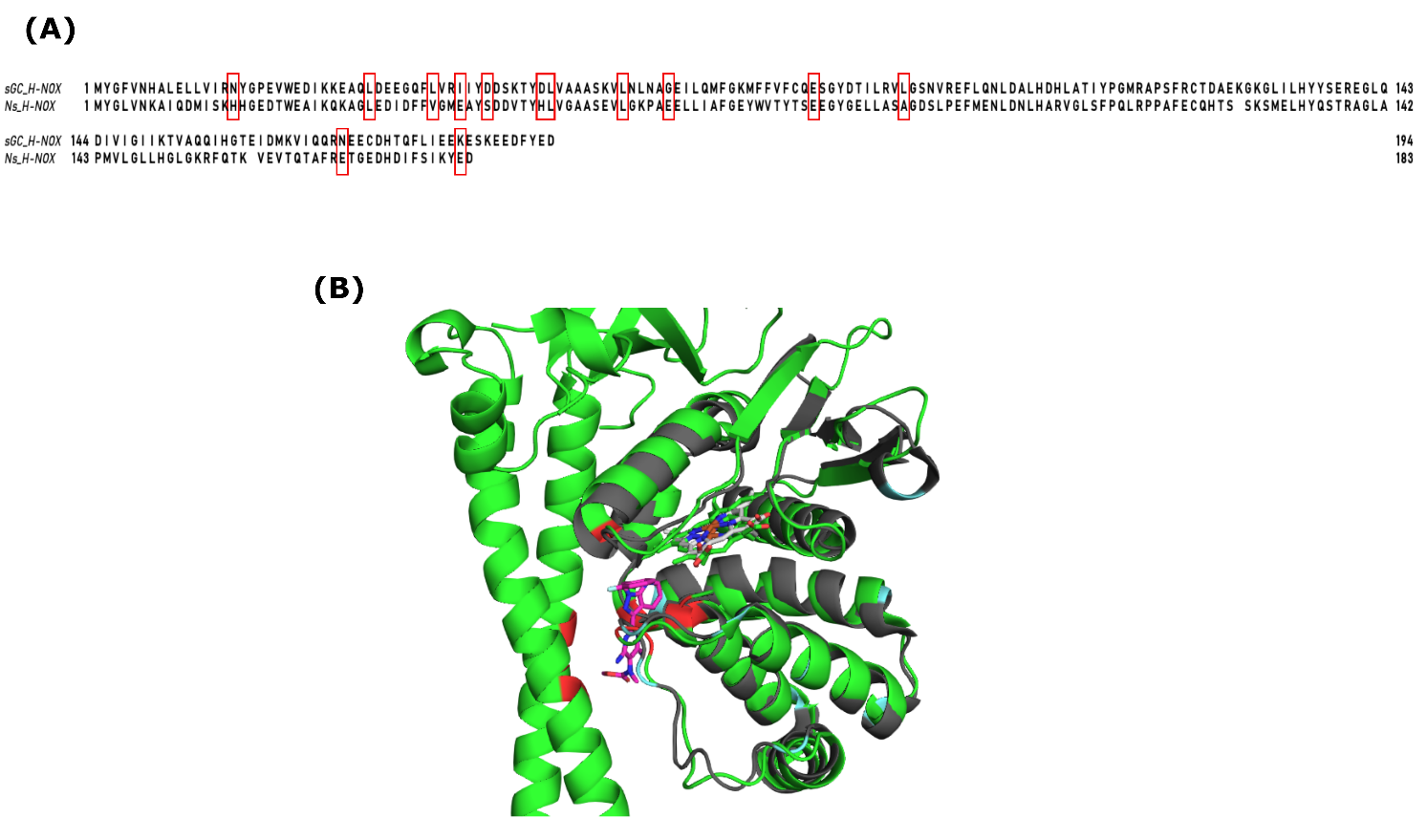


**Supplementary Figure 1.** **(A)** Sequence alignment of H-NOX domains from *Nostoc sp.* and *β*_1_ sGC **(B)** Structural alignment of the H-NOX domains of human sGC and *Nostoc sp.* (Kang et al., 2019)


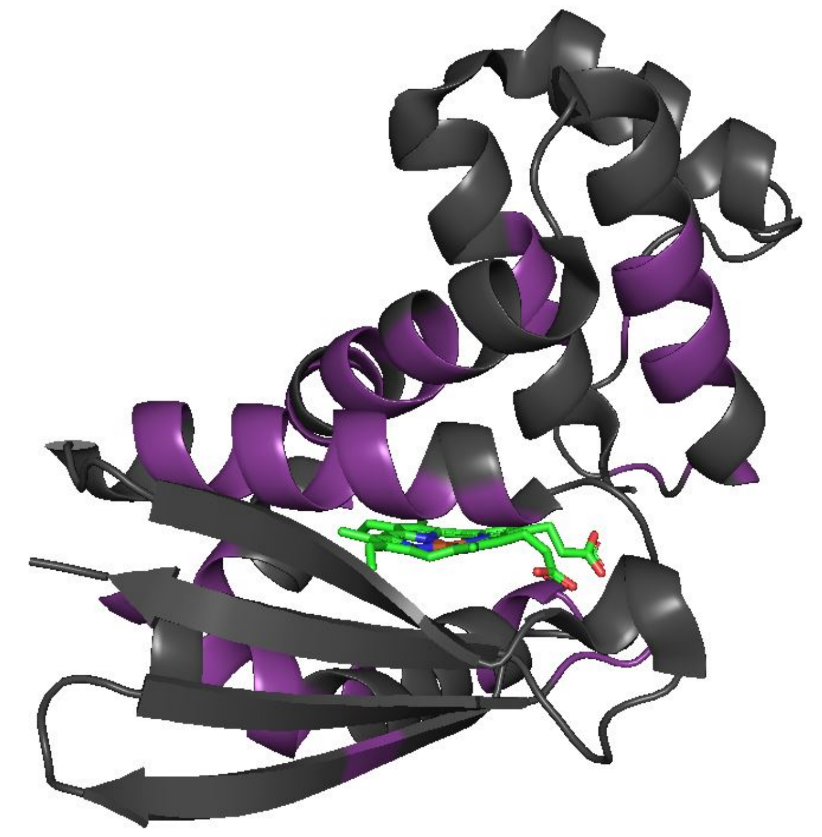


Supplementary Figure 2: Ribbon representation of the X-ray structure of *Ns* H-NOX domain (PDB id: 4IAM) (Kumar et al., 2013). The residues with CSPs after BAY 58-2667 addition above the threshold are mapped onto the surface using in purple (Argyriou et al., 2021)


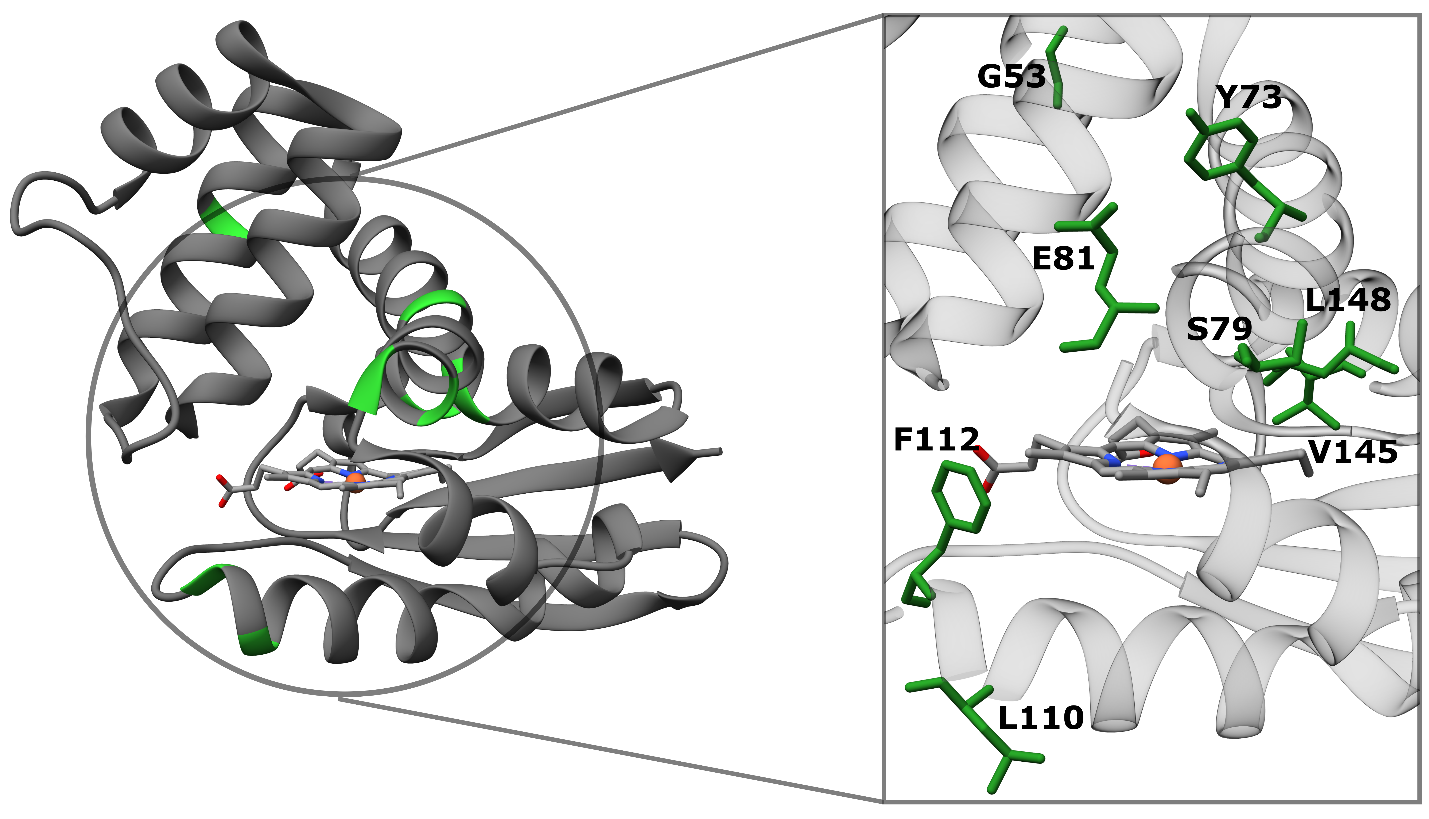


**Supplementary Figure 3.** Ribbon representation of X-ray structure of the heme bound *Ns* H- NOX. Residues with missing N-H after ODQ addition (1:0.5) are colored with green.


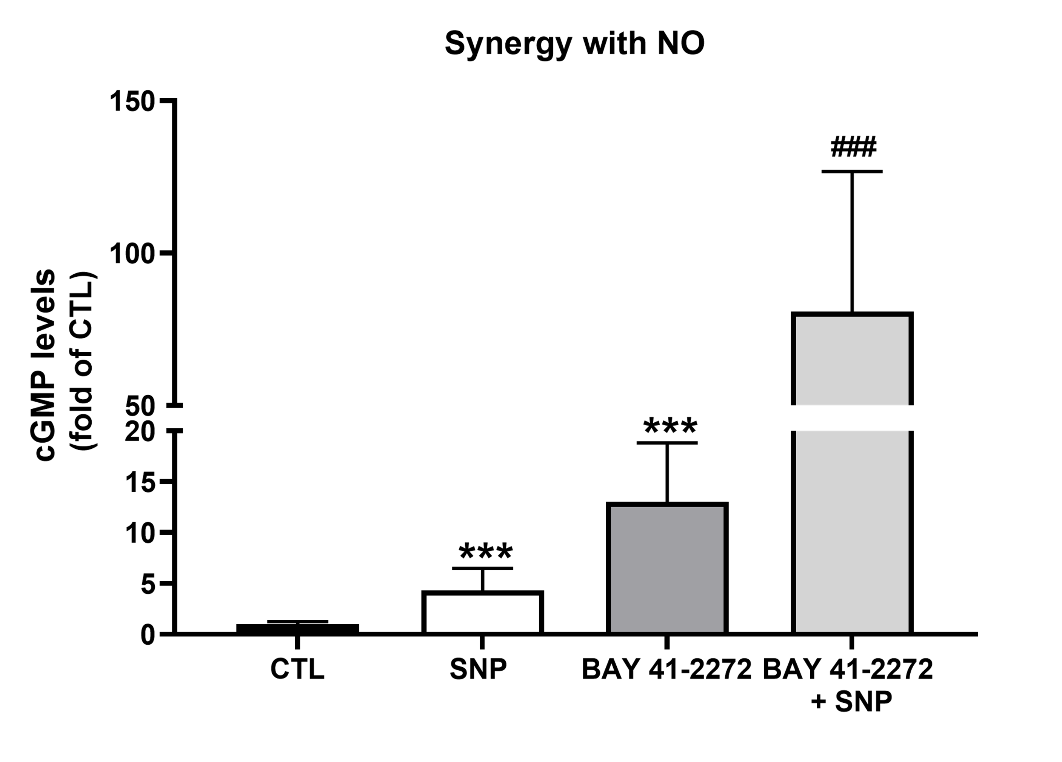


**Supplementary Figure 4.** BAY 41-2272 synergistic effect in A7R5 rat aortic smooth muscle (RaoSM) cells. Cells were exposed to 100μΜ SNP, 10μM of BAY 41-2272 or vehicle (DMSO). 15 minutes later, the extracts were taken, analyzed for cGMP levels and normalized for total protein content. n = 6 determinations obtained in 3 independent experiments. Data are expressed as mean ± STDEV of fold-cGMP levels (pmol/mg protein) compared to CTL (vehicle-treated) cells. P values were determined by 2-tailed Student’s t-test. ***: p<0.001 vs CTL, ###: p<0.001 vs SNP or BAY 41-2272 alone.

**Supplementary Table 1: K_D_ ^BAY 41-2272^ values estimated from NMR Chemical Shift Perturbation**

| **Amino acid** | **K_D_** | **Δδ_max_** | **Amino acid** | **K_D_** | **Δδ_max_** |
| --- | --- | --- | --- | --- | --- |
| H16 | 1.42mM ± 0.64 | 0.028 ± 0.0061 | E64 | 1.95mM ± 0.61 | 0.042 ± 0.0073 |
| A29 | 1.30mM ± 0.47 | 0.027 ± 0.0046 | L66 | 1.42mM ± 0.44 | 0.037 ± 0.0058 |
| G30 | 1.44mM ± 0.40 | 0.033 ± 0.0045 | E80 | 3.6 ± 1.3 | 0.026 ± 0.0074 |
| V38 | 1.86mM ± 0.45 | 0.045 ± 0.006 | L115 | 1.23 ± 0.59 | 0.030 ± 0.0058 |
| G39 | 1.31mM ± 0.51 | 0.027 ± 0.0051 | L141 | 1.13 ± 0.41 | 0.029 ± 0.0049 |
| E41 | 1.37 ± 0.71 | 0.029 ± 0.0074 | H150 | 1.76 ± 0.98 | 0.029 ± 0.0087 |
| H50 | 2.2mM ± 0.4 | 0.039 ± 0.0088 | F156 | 1.47 ± 0.40 | 0.026 ± 0.0035 |
| L51 | 1.47mM ± 0.42 | 0.034 ± 0.005 | E169 | 1.56 ± 0.86 | 0.029 ± 0.0083 |

Argyriou, A. I., Makrynitsa, G. I., Dalkas, G., Georgopoulou, D. A., Salagiannis, K., Vazoura, V., et al. (2021). Replacement of heme by soluble guanylate cyclase (sGC) activators abolishes heme-nitric oxide/oxygen (H-NOX) domain structural plasticity. *Curr. Res. Struct. Biol.* 3, 324–336. doi:10.1016/J.CRSTBI.2021.11.003.

Kang, Y., Liu, R., Wu, J. X., and Chen, L. (2019). Structural insights into the mechanism of human soluble guanylate cyclase. *Nature* 574 (7777), 206–210. doi:10.1038/s41586-019-1584-6.

Kumar, V., Martin, F., Hahn, M. G., Schaefer, M., Stamler, J. S., Stasch, J. P., et al. (2013). Insights into BAY 60-2770 activation and S -nitrosylation-dependent desensitization of soluble guanylyl cyclase via crystal structures of homologous Nostoc H-NOX domain complexes. *Biochemistry* 52 (20), 3601–3608. doi:10.1021/bi301657w.
